# Normalization of magnesium deficiency attenuated mechanical allodynia, depressive-like behaviors, and memory deficits associated with cyclophosphamide-induced cystitis by inhibiting TNF-α/NF-κB signaling in female rats

**DOI:** 10.1186/s12974-020-01786-5

**Published:** 2020-04-02

**Authors:** Jia-Liang Chen, Xin Zhou, Bo-Long Liu, Xu-Hong Wei, Hong-Lu Ding, Zhi-Jun Lin, Hai-Lun Zhan, Fei Yang, Wen-Biao Li, Jun-Cong Xie, Min-Zhi Su, Xian-Guo Liu, Xiang-Fu Zhou

**Affiliations:** 1 Department of Urology, The Third Affiliated Hospital of Sun Yat-sen University, 600 W Tianhe Rd, Guangzhou, 510630 China; 2 Pain Research Center and Department of Physiology, Zhongshan School of Medicine, Sun Yat-sen University, 74 Zhongshan Rd. 2, Guangzhou, 510080 China; 3 Guangdong Provincial Key Laboratory of Brain Function and Disease, 74 Zhongshan Rd. 2, Guangzhou, 510080 China; 4 Department of Rehabilitation, The Third Affiliated Hospital and Lingnan Hospital of the Sun Yat-sen University, 2693 Kaichuang Rd, Guangzhou, 510700 China

**Keywords:** Bladder pain syndrome, Cystitis, Neuroinflammation, Depression, Memory dysfunction

## Abstract

**Background:**

Bladder-related pain symptoms in patients with bladder pain syndrome/interstitial cystitis (BPS/IC) are often accompanied by depression and memory deficits. Magnesium deficiency contributes to neuroinflammation and is associated with pain, depression, and memory deficits. Neuroinflammation is involved in the mechanical allodynia of cyclophosphamide (CYP)-induced cystitis. Magnesium-L-Threonate (L-TAMS) supplementation can attenuate neuroinflammation. This study aimed to determine whether and how L-TAMS influences mechanical allodynia and accompanying depressive symptoms and memory deficits in CYP-induced cystitis.

**Methods:**

Injection of CYP (50 mg/kg, intraperitoneally, every 3 days for 3 doses) was used to establish a rat model of BPS/IC. L-TAMS was administered in drinking water (604 mg·kg^−1^·day^−1^). Mechanical allodynia in the lower abdomen was assessed with von Frey filaments using the up-down method. Forced swim test (FST) and sucrose preference test (SPT) were used to measure depressive-like behaviors. Novel object recognition test (NORT) was used to detect short-term memory function. Concentrations of Mg^2+^ in serum and cerebrospinal fluid (CSF) were measured by calmagite chronometry. Western blot and immunofluorescence staining measured the expression of tumor necrosis factor-α/nuclear factor-κB (TNF-α/NF-κB), interleukin-1β (IL-1β), and *N*-methyl-d-aspartate receptor type 2B subunit (NR2B) of the *N*-methyl-d-aspartate receptor in the L6–S1 spinal dorsal horn (SDH) and hippocampus.

**Results:**

Free Mg^2+^ was reduced in the serum and CSF of the CYP-induced cystitis rats on days 8, 12, and 20 after the first CYP injection. Magnesium deficiency in the serum and CSF correlated with the mechanical withdrawal threshold, depressive-like behaviors, and short-term memory deficits (STMD). Oral application of L-TAMS prevented magnesium deficiency and attenuated mechanical allodynia (*n* = 14) and normalized depressive-like behaviors (*n* = 10) and STMD (*n* = 10). The upregulation of TNF-α/NF-κB signaling and IL-1β in the L6–S1 SDH or hippocampus was reversed by L-TAMS. The change in NR2B expression in the SDH and hippocampus in the cystitis model was normalized by L-TAMS.

**Conclusions:**

Normalization of magnesium deficiency by L-TAMS attenuated mechanical allodynia, depressive-like behaviors, and STMD in the CYP-induced cystitis model via inhibition of TNF-α/NF-κВ signaling and normalization of NR2B expression. Our study provides evidence that L-TAMS may have therapeutic value for treating pain and comorbid depression or memory deficits in BPS/IC patients.

## Background

Depression and memory deficits are two serious comorbidities of chronic pain. A community population-based research study revealed that approximately one third of participants with chronic pain also had comorbid depression [1]. About two thirds of patients with chronic pain also have memory dysfunction [2]. Bladder pain syndrome/interstitial cystitis (BPS/IC) is a chronic condition where urinary bladder-related pain is a symptom that impacts a patient’s life. Population-based studies have shown that the female-to-male ratio ranges from 5:1 [3] to 10:1 [4], and most clinical studies include women only since confounding diagnoses are common in men with chronic prostatitis [5]. Additionally, depression and memory problems often accompany and amplify the symptoms of BPS/IC [6]. A previous study revealed that about one third of these patients have depressive disorders [7]. More female patients suffer from depression than males [8]. A population-based study also showed that patients with BPS/IC were more likely to have memory problems than healthy people [9], and patients with BPS/IC with depression report increased pain scores [7]. However, there are limited satisfying treatment options presently available to improve both the pain symptoms and the comorbidities of BPS/IC. Moreover, the mechanisms underlying the comorbidity of bladder-related pain and depression/memory dysfunction in BPS/IC are not clearly identified. The clarification of such mechanisms is complex, but the exploration of factors that can link the comorbidities is easier and also of great clinical value.

It has been suggested that magnesium deficiency is associated with neuropathic pain [10] and induces nociception sensitization in normal animals [11, 12]. Moreover, magnesium deficiency is also correlated with the depressive symptoms induced by fibromyalgia [13] (a pain syndrome often accompanied with BPS/IC) and the impaired memory function associated with neuropathic pain [14]. However, whether magnesium deficiency is associated with bladder-related pain and comorbid depression or memory dysfunction is still unknown.

Inflammatory mechanisms, especially neuroinflammation, are believed to play an important role in the development of pain, depression, and memory deficits [15–17]. Our previous work demonstrated that neuroinflammation participates in the initiation and maintenance of mechanical allodynia in cyclophosphamide (CYP)-induced cystitis [18, 19]. Moreover, intrathecal (i.t.) injection of IL-1ra (interleukin-1 receptor antagonist) significantly attenuated the mechanical allodynia induced by CYP-induced cystitis [19]. However, whether neuroinflammation is also associated with depression or memory deficits in the cystitis model and whether inhibition of neuroinflammation influences these two comorbidities are still unknown.

Taken together, magnesium deficiency and neuroinflammation are two underlying therapeutic targets to treat pain and comorbid depression and memory deficits. Interestingly, it has been reported that magnesium deficiency potentiates a generalized inflammatory state [20] and also evokes neuroinflammation leading to depressive-like behavior [21]. Supplementation of magnesium may inhibit such an inflammatory response, especially the neuroinflammatory component. As a novel magnesium compound, magnesium-L-Threonate (L-TAMS) has been found to be able to infiltrate through the blood-brain barrier and elevate brain magnesium levels following oral application [22]. Previous research showed that L-TAMS could improve memory function in Alzheimer’s disease (AD) through inhibition of neuroinflammation [23]. Additionally, L-TAMS was able to inhibit neuroinflammation, leading to attenuation of neuropathic pain as well as neuropathic pain-associated memory deficits [14, 24]. However, no study has shown whether and how L-TAMS influences bladder-related pain and the accompanying depressive symptoms and memory deficits in BPS/IC.

Based on the above evidence, we hypothesize that L-TAMS could have a therapeutic effect on mechanical allodynia and on comorbid depression and memory deficits in the CYP-induced cystitis model. This study aimed to investigate whether and how oral application of L-TAMS could attenuate the symptoms described above in the CYP-induced cystitis model.

## Methods

### Animals

Female Sprague Dawley rats (200–220 g) were obtained from the Institute of Experimental Animals of Sun Yat-sen University, Guangzhou, China. The animals were housed in a temperature- and humidity-controlled room (24 ± 1 °C) under a 12/12-h light/dark cycle (06:00–18:00 h). Animals had access to food and water ad libitum. All experimental procedures were approved by the Animal Care Committee of Sun Yat-sen University and conducted in accordance with the guidelines of the National Institutes of Health on animal care and ethical guidelines.

### Drug administration

**Fig. 1:**
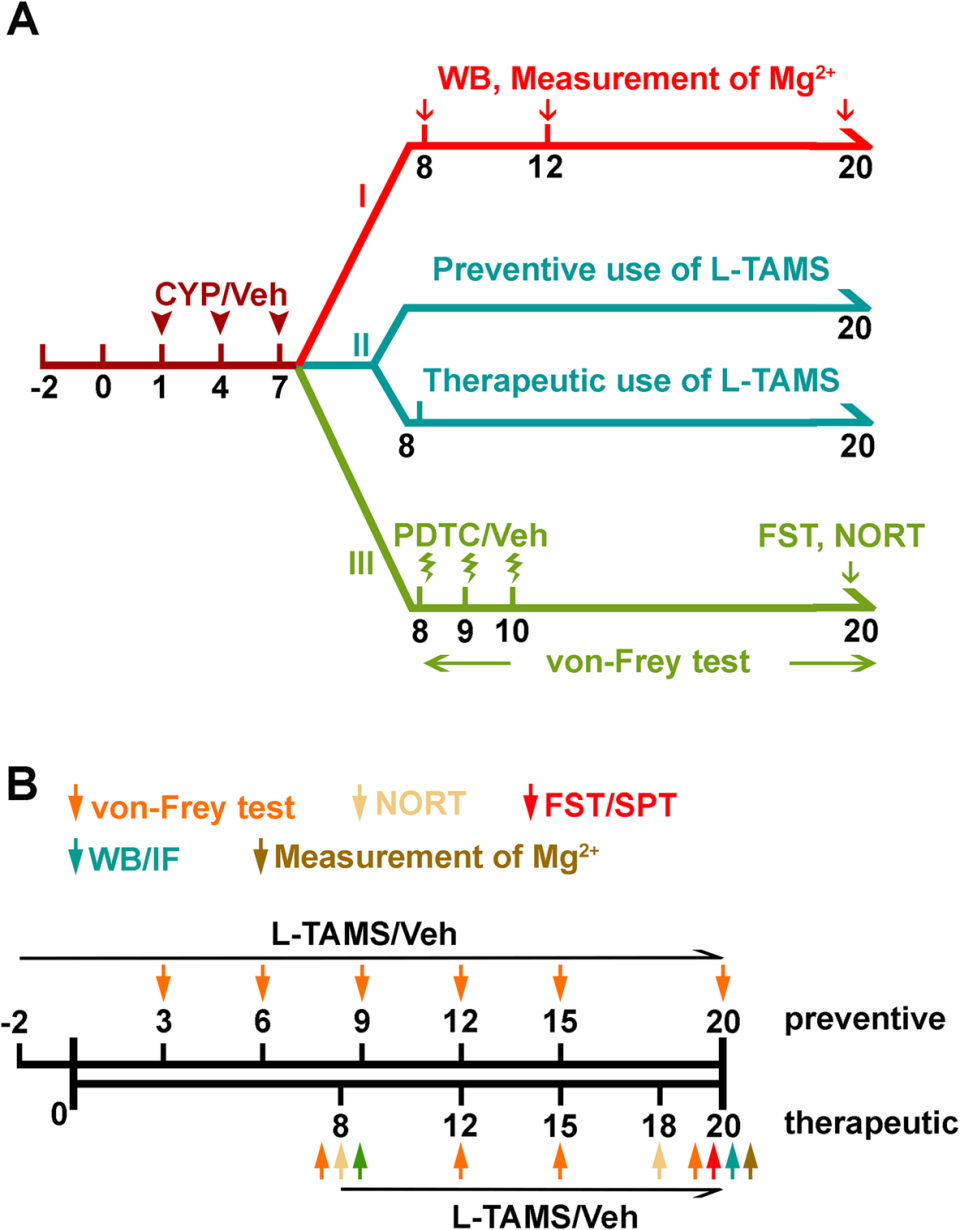
Flowchart of the experimental procedure. **a** The study consisted of three parts. In part I, western blot analyses were performed at the three time points, day 8, day 12, and day 20, to determine the expression level of TNF-α/NF-κB, IL-1β, and NR2B in CYP-induced cystitis animals. The concentration of Mg^2+^ was also measured at the three time points. In part II, after treatment with L-TAMS, behavioral tests and molecular experiments were performed to investigate the effect of L-TAMS in the CYP-treated animals. The details in part II were shown in **b**. Additionally, in part III, the effect of inhibiting NF-κB signaling was determined by behavioral tests

CYP was used to establish the cystitis model as previously described [18]. Briefly, CYP (25 mg/kg; Sigma, St Louis, MO) was intraperitoneally (i.p.) injected every 3 days for 3 doses. Oral application of L-TAMS (Neurocentria Inc., USA) was used as a method of supplementing magnesium. L-TAMS was administered in drinking water (604 mg∙kg ^−1^∙day ^−1^) [22, 24] along with 50 mg∙kg^−1^∙day^−1^ of elemental magnesium over the course of the study. L-TAMS was applied over two different time periods as shown in Fig. 1b and Fig. 3a, b: one was initiated 3 days prior to the first CYP dose while the other was initiated 1 day post the last CYP injection.

### Experimental design

The flowchart for experimental design is provided in Fig. 1, and the number of animals used in each test was outlined in Supplemental Table 1. We designed three experimental parts for our present study. In part I, four animal groups were included: the Veh group as a control that was saline injected i.p., while the other three groups, CYP d8, CYP d12, and CYP d20, were CYP-treated and anesthetized for western blot sample harvest on days 8, 12, and 20, respectively, after the first CYP injection. The magnesium concentration changes were evaluated in serum and cerebrospinal fluid (CSF) at these three time points. In addition, the correlation between Mg^2+^ and bladder-related pain or comorbidities was also assessed. Moreover, the expression changes in TNF-α/NF-κB and related factors, including interleukin-1beta (IL-1β) and *N*-methyl-d-aspartate (NMDA) receptor type 2B (NR2B), were also assessed using western blot analysis.

Part II of the study helped us to understand whether and how magnesium deficiency influences bladder-related pain and comorbidities, as we performed behavioral evaluation to explore whether normalization of magnesium deficiency with L-TAMS can attenuate bladder-related pain and comorbid depressive symptoms (d20), or memory deficits (d18) in the CYP-induced cystitis rat model. In this part, four animal groups were included: the Veh group with drinking water, the L-TAMS group having normal animals with L-TAMS, the CYP group with drinking water, and the CYP + L-TAMS group having cystitis animals with L-TAMS. To avoid mutual influence among the different behavioral tests, each group only received one behavioral test and then anesthetized on day 20 for Mg^2+^ measurements. Additionally, on day 20, tissue samples for western blot analysis and immunofluorescence to measure the expression change of TNF-α/NF-κB, IL-1β, and NR2B were harvested from animals that received the von Frey test.

Last, we set up part III of the study to verify whether inhibition of NF-κB signaling with pyrrolidinedithiocarbamate ammonium (PDTC) can directly alleviate bladder-related pain as well as the comorbid depressive-like symptoms and memory deficits in the CYP-induced cystitis animal model. Four groups were included in this part: the Veh group with DMSO, the PDTC group having normal animals with PDTC, the CYP group, and the CYP + PDTC group having cystitis animals with PDTC.

### Intrathecal injection

Intrathecal injection (i.t.) was performed as described previously [25]. Under anesthesia, a 25-G needle connected to a 25-μl Hamilton syringe was inserted percutaneously into the vertebral canal between L5 and L6. A tail-flick reaction denoted a successful puncture. A rat was injected i.t. with 20 μl of 1% Chicago sky blue (C8678, Sigma-Aldrich) to determine whether the drug injected i.t. was able to reach the L6–S1 spinal cord level. After that, a dose of 200 ng [26] of the NF-κВ signaling inhibitor PDTC was injected i.t. for 3 consecutive days after the last CYP injection.

### Measurement of mechanical withdrawal threshold

Since bladder-related pain is difficult to assess directly, measurement of the lower abdominal withdrawal threshold was used as a substitute for assessment of bladder-related pain [27]. The up-down method, as described previously [28], and a series of von Frey filaments (rated at 0.4, 0.6, 1, 2, 4, 6, 8, 15 g) were used to measure evoked pain in the lower abdomen, the area of referred pain associated with vesical pathologies. Animals were acclimatized to the chamber environment for 30 min every day for 2 days before testing, and those with an abnormal basic pain threshold were excluded. Different lower abdominal areas were stimulated to avoid desensitization. The 2-g stimulus in the middle of the series was applied first. Each stimulus consisted of a 6–8-s application of the von Frey filament to the lower abdominal region in at least 5-min intervals. A stronger or weaker filament was applied after a negative or positive response, respectively, was elicited. “Licking behavior” and “freezing behavior” were considered positive responses.

### Behavioral experiments

*Forced swim test* (FST) was used to detect depressive-like behavior as previously described [29] with slight modifications. Rats were placed individually in a transparent glass cylinder tank (50 cm height × 30 cm in diameter) filled with water to 45 cm depth at 22–25 °C. The tank was thoroughly cleaned before testing, and the water was changed after each test. The day prior to the test, rats were placed into the tank and swam for 15 min. On the test day, swimming behavior was assessed for 6 min and the immobility time (floating and treading water just enough to keep the head above water) was recorded during the last 4 min.

*Sucrose preference test* (SPT) was also carried out to determine depressive-like behaviors. The test was performed as previously described [30]. Briefly, rats were singly housed and trained to drink 2% sucrose solution in place of water for 2 days. After that, rats were deprived of water for 24 h and then underwent a 2-h test, during which they were exposed to one bottle of water and one bottle of 2% sucrose solution. Additionally, during the 2-h test, the positions of the two bottles were switched at the 1-h time point. Total consumption of each fluid was then measured, and the sucrose consumption ratio was calculated as a ratio of the total consumption of sucrose over the total consumption of both water and sucrose.

*Novel object recognition test* (NORT) was used to determine short-term memory ability, as previously described [15]. Before the test, each rat was acclimated to the opaque box (60 × 60 × 40 cm) for 10 min each day for two consecutive days. The test was divided into two sections. In the sample phase, each rat was exposed to two different objects in the box for 5 min. After a 10-min retention interval, the less explored object of the two was replaced by a new one and the rat was placed back in the box and exposed to two objects (the familiar one and a novel one) for a further 5-min “acquisition phase.” An experimenter blinded to the identity of the tested rats then measured the time spent exploring each object. The recognition index reflecting the short-term memory ability was set as the percent time spent exploring the novel object.

### Measurement of extracellular free magnesium

The calmagite chronometry method [31] was used to measure the concentration of free Mg^2+^ in serum and CSF of rats. The blood samples were collected from the orbital sinus and then centrifuged at 2000 rpm for 10 min to isolate serum, and CSF was collected from the cisterna magna.

### Western blot analysis

We used a modified western blot protocol [32] in our present study. Rats were anesthetized with sodium pentobarbital (50 mg/kg, i.p.). The L6–S1 spinal dorsal horn (SDH) and hippocampus were quickly harvested, and the supernatant was collected and then frozen at − 80 °C after the tissue samples were mechanically homogenized and centrifuged. Samples were homogenized in RIPA lysis buffer containing proteinase and phosphatase inhibitors. The bicinchoninic acid method was used to measure the protein concentration of the supernatant. Proteins in the supernatant were separated by sodium dodecyl sulfate-polyacrylamide gel electrophoresis and then transferred onto polyvinylidene fluoride membranes (Millipore, Billerica, MA, USA). The membranes were blocked in 5% bovine serum albumin solution for 60–90 min at 37 °C. After blocking, the blots were incubated with primary antibody (reference/RRID shown in Supplemental Table 2) for TNF-α (1:1000; Bioworld Technology, Inc., Louis Park, MN, USA), phospho-p65 (*p*-p65, Ser311, 1:1000; Affinity Biosciences, OH, USA), p65 (1:1000; Abcam, Cambridge, UK), IL-1β (1:2500; Abcam), NR2B (1:1000; Abcam), and β-actin (1:1000; Cell Signaling Technology, Danvers, MA) overnight at 4 °C. Secondary antibodies conjugated with horseradish peroxidase (1:10,000; KPL, SeraCare, Milford, MA, USA) were applied, and the membrane was incubated for 1 h. Immune complexes were detected with an enhanced chemiluminescence liquid (Millipore). A computer-assisted imaging analysis system (ImageJ; National Institutes of Health, Bethesda, MD, USA) was used to quantify the band intensities.

### Immunofluorescence

Immunofluorescence was performed as described in a previous study [33]. Under sodium pentobarbital anesthesia (50 mg/kg, i.p.), rats were perfused with 4% paraformaldehyde through the ascending aorta. The L6–S1 spinal cord section and brain were removed and post-fixed in paraformaldehyde for 30 min. The spinal cord and brain were then transferred to 30% sucrose for dehydration at 4 °C. Tissues underwent sectioning (25-μm thickness) and were processed for immunofluorescence staining. Sections were blocked for 1 h and then incubated with primary antibodies against TNF-α (1:200; Bioworld), *p*-p65 (1:100; Affinity), IL-1β (1:500; Abcam), neuronal nuclei (NeuN, 1:200; Millipore), glial fibrillary acidic protein (GFAP, 1:400; CST), and CD11b (OX-42, 1:400; Abcam) overnight at 4 °C. After incubation, the sections were incubated in Cy3 or Alexa-488 (Jackson Laboratories, Bar Harbor, ME, USA) conjugated secondary antibodies for 1 h at room temperature. A Leica fluorescence microscope (Leica DFC350 FX camera; Wetzlar, Germany) was used to measure and image the stained section. To quantify TNF-α, *p*-p65, and IL-1β expression in the L6–S1 SDH and hippocampus, the fluorescence intensity of each area was analyzed with ImageJ software. To verify the specificity of the antibodies used in our study, immunostaining was also performed in the same way but without primary antibodies. As shown in Supplemental Figure 1, the specificity of the antibody for *p*-p65 (S311) was identified by pre-absorption with blocking peptide.

### Statistical analysis

All data are expressed as mean ± standard error of the mean (SEM). SPSS 21.0 (SPSS, Inc., Chicago, IL, USA) was used to perform data analyses. The FST, SPT, NORT (Fig. 7c), western blot, immunofluorescence, and Mg^2+^ data were analyzed with a one-way analysis of variance (ANOVA) followed by the Tukey post hoc test for comparisons of more than three groups. For the mechanical withdrawal threshold and NORT (Fig. 3e), the data were statistically analyzed using a repeated-measures two-way ANOVA followed by a Tukey post hoc test. The Shapiro-Wilk test was used to verify normal distribution of the data before each ANOVA test. Linear regression analysis was performed, and the correlation coefficients were calculated to determine the relationship between Mg^2+^ and mechanical withdrawal threshold or floating time or recognition index. Differences with *P* < 0.05 were considered statistically significant.

## Results

### Magnesium deficiency in serum and CSF of the CYP-induced cystitis model was reversed by L-TAMS

**Fig. 2:**
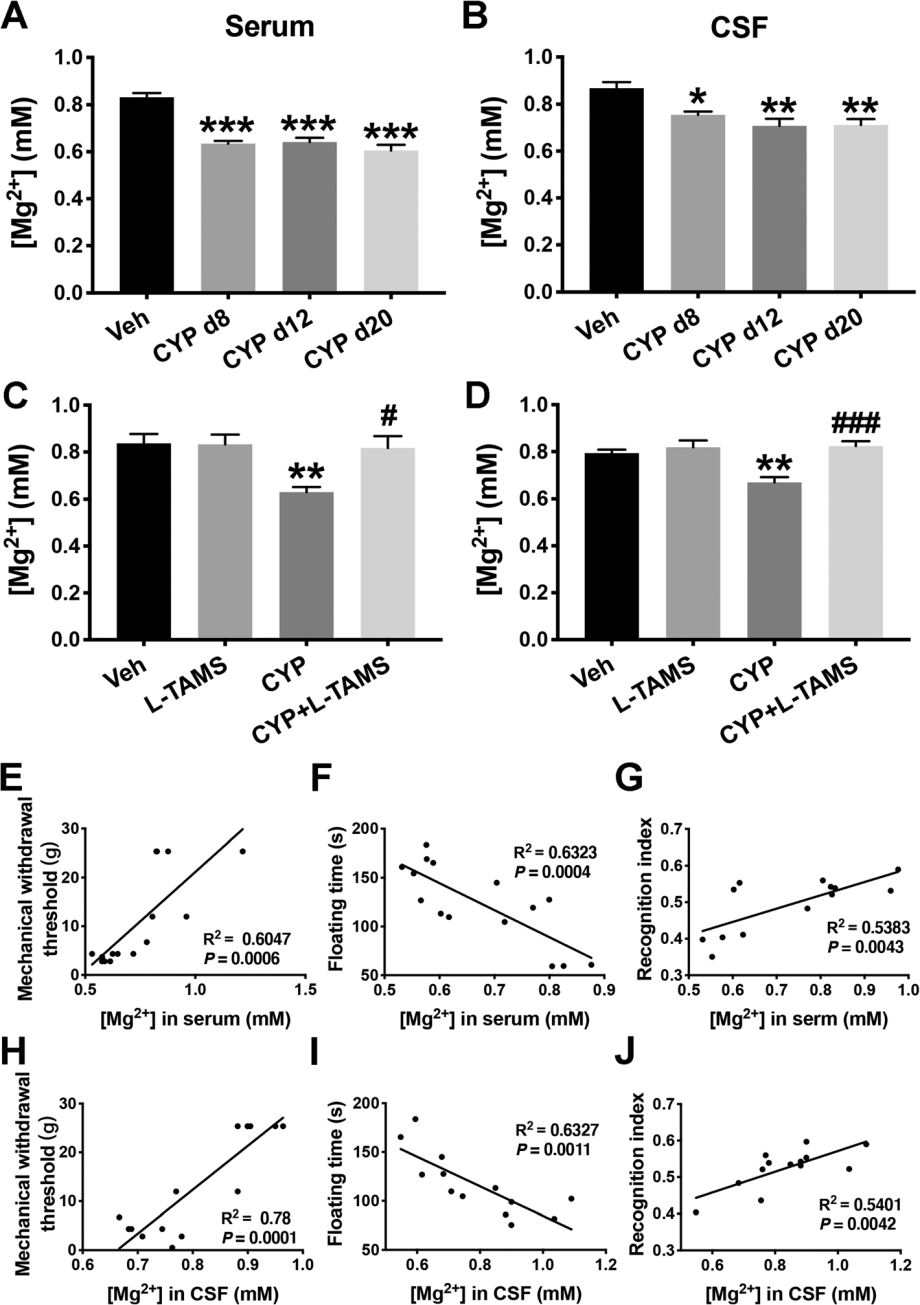
Decreased Mg^2+^ in the serum and CSF of CYP-induced cystitis animals was reversed by L-TAMS treatment. Free concentrations of Mg^2+^ in the serum (**a**) and CSF (**b**) of CYP-induced cystitis animals were assessed on day 8, day 12, and day 20 after the first CYP injection. *n* = 7 per group for serum and *n* = 5 per group for CSF. **c**, **d** After treatment with L-TAMS or vehicle, measurement of Mg^2+^ in cystitis animals was also performed on day 20. *n* = 10 per group for serum and *n* = 9 per group for CSF. **P < 0.05, **P < 0.01, and ***P < 0.001 vs. Veh group. ^#^*P* < 0.05 and ^###^*P* < 0.001 vs. CYP group. The data were analyzed by one-way ANOVA followed by the Tukey post hoc test. Concentrations of Mg^2+^ in the serum (**e**–**g**) and CSF (**h**–**j**) of cystitis rats were correlated with floating time in the FST and the short-term memory index in the NORT. *n* = 13 to 15 per group. The data in **e**–**j** were analyzed by linear regression, and the correlation coefficients were calculated

As shown in Fig. 2a–d, Mg^2+^ concentrations in both the serum and CSF were reduced at the three time points, day 8, day 12, and day 20, after the first CYP injection. Furthermore, oral application of L-TAMS reversed the reduction in Mg^2+^ concentrations and returned them to basal levels (*F*_(3, 36)_ = 6.23, *P* < 0.05 vs. CYP group for serum; *F*_(3, 36)_ = 10.39, *P* < 0.001 vs. CYP group for CSF) after 12 days of treatment, as measured on day 20 after the first injection. Additionally, L-TAMS did not affect the Mg^2+^ level in either the serum or the CSF of normal rats.

We next performed a linear regression analysis to explore the relationship between Mg^2+^ level and mechanical withdrawal threshold, as well as the relationship between Mg^2+^ level and floating time in the FST or recognition index in NORT. The results indicate that the concentrations of Mg^2+^ in serum and CSF were both positively correlated with the mechanical withdrawal threshold (Fig. 2e, h) and recognition index (Fig. 2f, i), whereas there were negative correlations with floating time (Fig. 2g, j).

### Reversal of magnesium deficiency with L-TAMS leads to attenuation of mechanical allodynia, depressive-like behaviors, and short-term memory deficits (STMD)

**Fig. 3:**
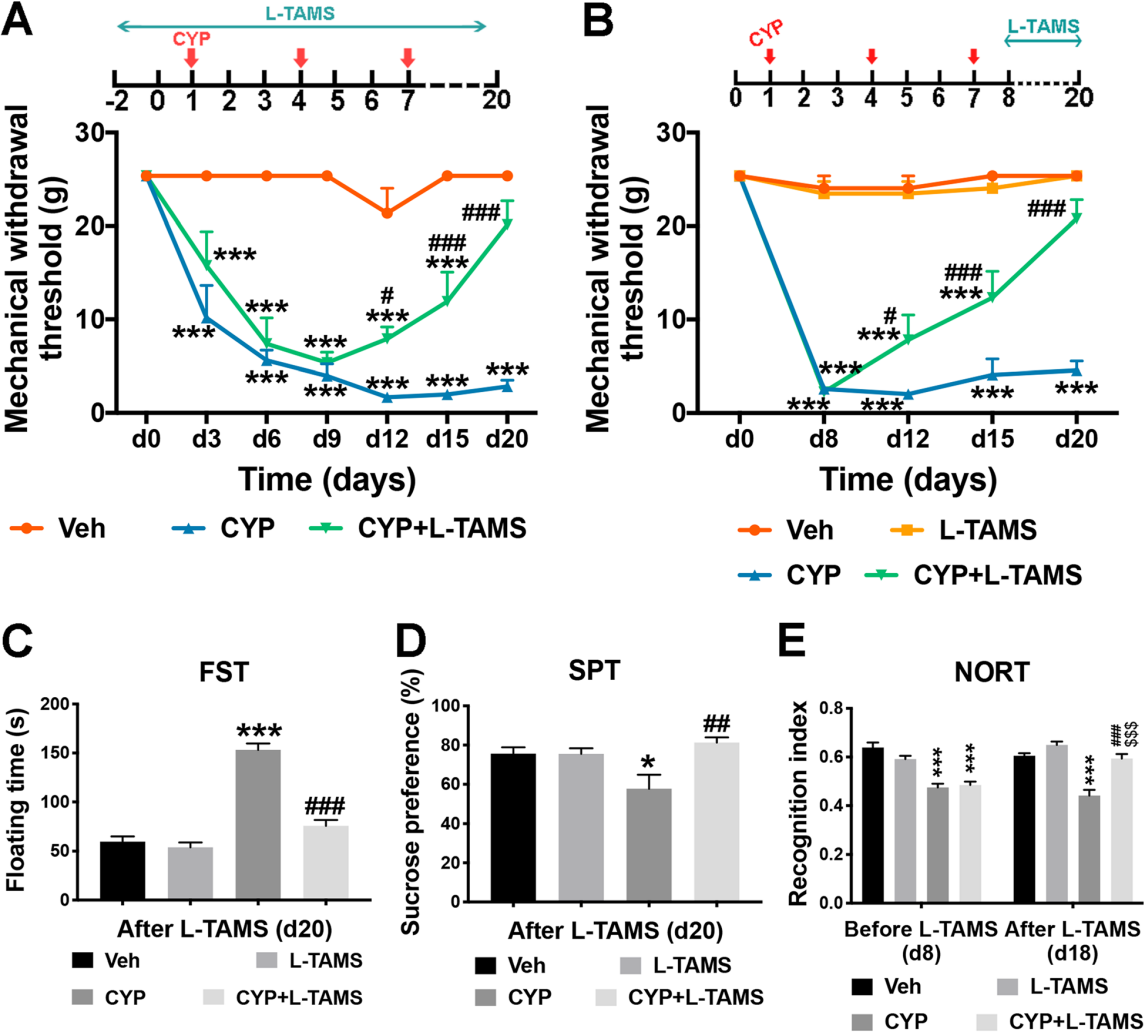
L-TAMS attenuates mechanical allodynia and comorbid depressive-like behaviors or memory deficits in CYP-induced cystitis animals. **a** L-TAMS was administered 3 days before the first dose of CYP and maintained until the end of the experiment. The mechanical withdrawal threshold in the CYP + L-TAMS group significantly increased from day 12 when compared with the CYP group without L-TAMS treatment, and it returned to baseline level, which was not significantly different from the vehicle group. *n* = 8 per group. **b** Treatment with L-TAMS was initiated together with the first CYP injection and maintained until the day of tissue harvest. The change in threshold in the CYP + L-TAMS group was similar to that in **a**. *n* = 14 per group. The data in **a** and **b** were analyzed by two-way ANOVA followed by the Tukey post hoc test. **c**, **d** Forced swim test (FST) and sucrose preference test (SPT) were performed on day 20. *n* = 10 and 5 per group for FST and SPT, respectively. **e** Novel object recognition test (NORT) was performed on day 8 and day 18 (prior to and after treatment, respectively). *n* = 10 per group. The data in **c** and **d** were analyzed by one-way ANOVA followed by the Tukey post hoc test. The data in **e** were analyzed by two-way ANOVA followed by the Tukey post hoc test. ***P < 0.05 and ***P < 0.001 vs. Veh group. ^#^*P* < 0.05, ^##^*P* < 0.01, and ^###^*P* < 0.001 vs. CYP group. ^$$$^*P* < 0.001 vs. CYP + L-TAMS group prior to L-TAMS treatment, on day 8

To investigate the effect of L-TAMS on mechanical allodynia in the CYP-induced cystitis model, we designed two experiments: L-TAMS was administered pre- (Fig. 3a) and post-CYP-induced cystitis model establishment (Fig. 3b) to determine its preventive and therapeutic effect on mechanical allodynia, respectively (also shown in Fig. 1b). As shown in Fig. 3a, b, L-TAMS began to significantly attenuate the decrease in the mechanical withdrawal threshold in the cystitis model from day 12 after the first CYP injection (*F*_(2, 21)_ = 108.2, *P* < 0.05 vs. CYP group for “a”; *F*_(3, 52)_ = 107.6, *P* < 0.01 vs. CYP group for “b”). The thresholds in the CYP + L-TAMS group in the two experiments both gradually increased to the basal level from day 12. On day 20, the difference in the threshold between the CYP + L-TAMS and vehicle groups was no longer significant. We also found that the application of L-TAMS did not affect the mechanical withdrawal threshold of normal rats.

In addition, the effect of L-TAMS on depressive-like behavior and short-term memory ability was determined using the FST, SPT, and NORT, respectively. Considering that FST itself is a test that can influence depressive-like behavior [34], we only performed the test once. The FST performed on day 20 shows that the floating time in the cystitis rats was significantly longer than that of the vehicle rats (*P* < 0.001). Moreover, the cystitis group that received L-TAMS treatment showed a shorter floating time than those without L-TAMS treatment (*F*_(3, 16)_ = 54.96, *P* < 0.001) (Fig. 3c). Additionally, the SPT result shown in Fig. 3d indicated that the reduced sucrose preference ratio present in the CYP-induced cystitis group also returned to basal level after L-TAMS treatment (*F*_(3, 16)_ = 5.46, *P* < 0.01 vs. CYP group). NORT was performed on day 8 (the day after the last CYP injection and prior to L-TAMS treatment) to determine the short-term memory ability in the cystitis group. No significant change was found in the two cystitis groups. However, after a 10-day treatment with L-TAMS, on day 18, the recognition index between the cystitis group with and without L-TAMS treatment became significantly different (*F*_(3, 36)_ = 41.71, *P* < 0.001). The recognition index of the cystitis model with L-TAMS treatment increased back to basal level on day 18 (Fig. 3e). Additionally, all the FST, PST, and NORT results in the normal animals were not affected by oral application of L-TAMS (Fig. 3c–e).

### TNF-α/NF-κB signaling and IL-1β were upregulated in the SDH and hippocampus of the CYP-induced cystitis model, and inhibition of NF-κB signaling attenuated mechanical allodynia, depressive-like behavior, and STMD

Upregulations of TNF-α/NF-κB signaling as well as the pro-inflammatory factor IL-1β in the spinal cord are important neuroinflammatory mechanisms in neuropathic pain [35]. Neuroinflammation initiated by TNF-α located at the hippocampus could be a common mechanism between pain and depression [36] or memory deficits [37]. However, the role of TNF-α/NF-κB signaling and IL-1β expression in the SDH and hippocampus of the CYP-induced cystitis model has not yet been clearly identified.

**Fig. 4:**
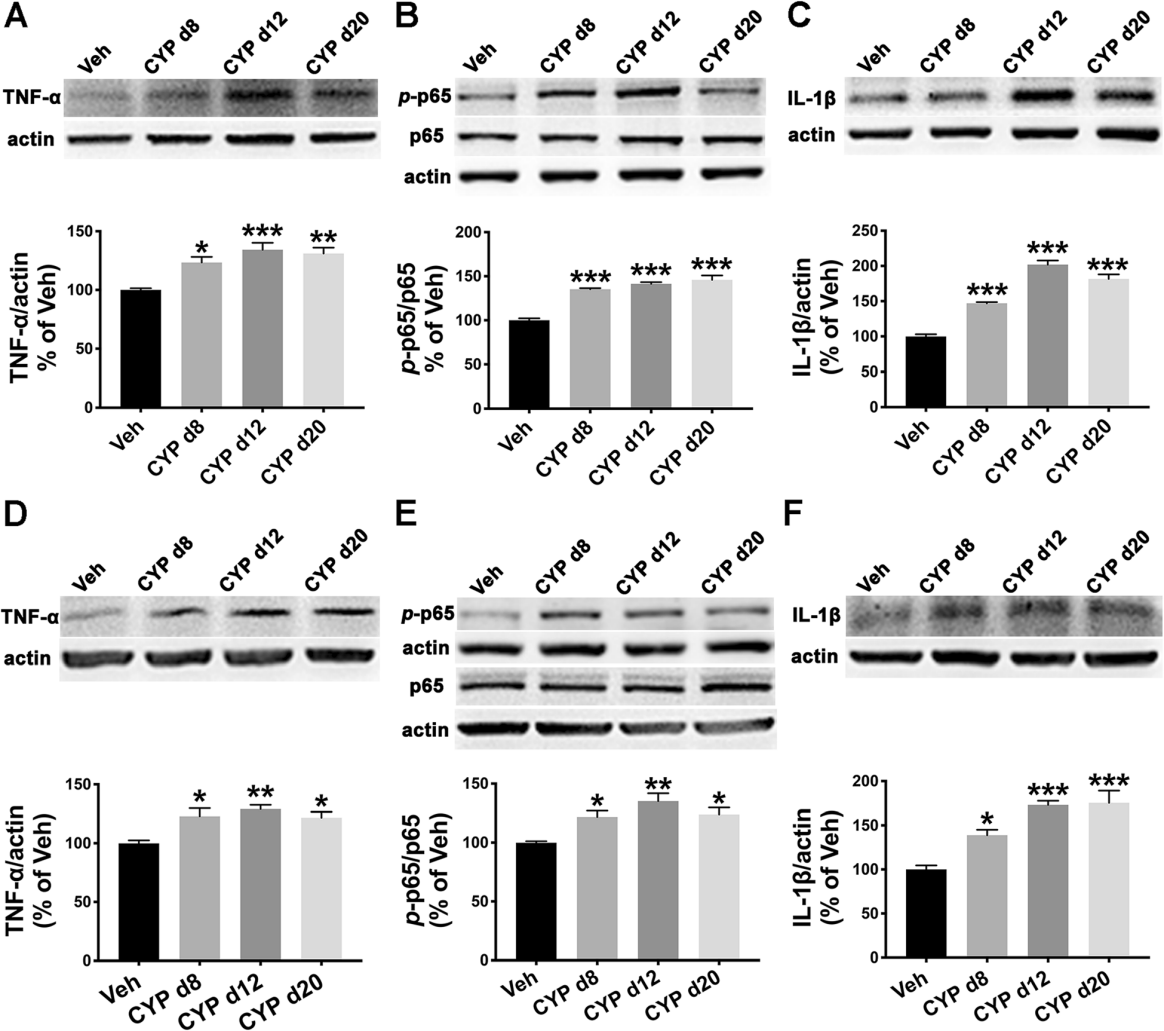
TNF-α/NF-κB signaling and IL-1β were upregulated in the SDH and hippocampus of the CYP-induced cystitis model. **a**–**c** Western blot analysis results indicate that TNF-α, the phospho-p65 (*p*-p65)/p65 ratio, and IL-1β were increased in the SDH of the CYP-induced cystitis model at all three time points (days 8, 12, and 20) after CYP injection. **d**–**f** TNF-α, the *p*-p65/p65 ratio, and IL-1β also showed higher expression in the hippocampus of the cystitis group than in the Veh group at the three time points. *P < 0.05, **P < 0.01, and ***P < 0.001 vs. Veh group. The data were analyzed by one-way ANOVA followed by the Tukey post hoc test

We performed western blot analyses to examine TNF-α/NF-κB signaling and IL-1β expression in the SDH and hippocampus harvested on days 8, 12, and 20. As shown in Fig. 4a–c, TNF-α, the *p*-p65/p65 ratio, and IL-1β were all significantly upregulated in the SDH at all three time points when compared with the vehicle group. Similar overexpression of the above factors was also found in the hippocampus of rats in the cystitis model group (Fig. 4d–f).

**Fig. 5:**
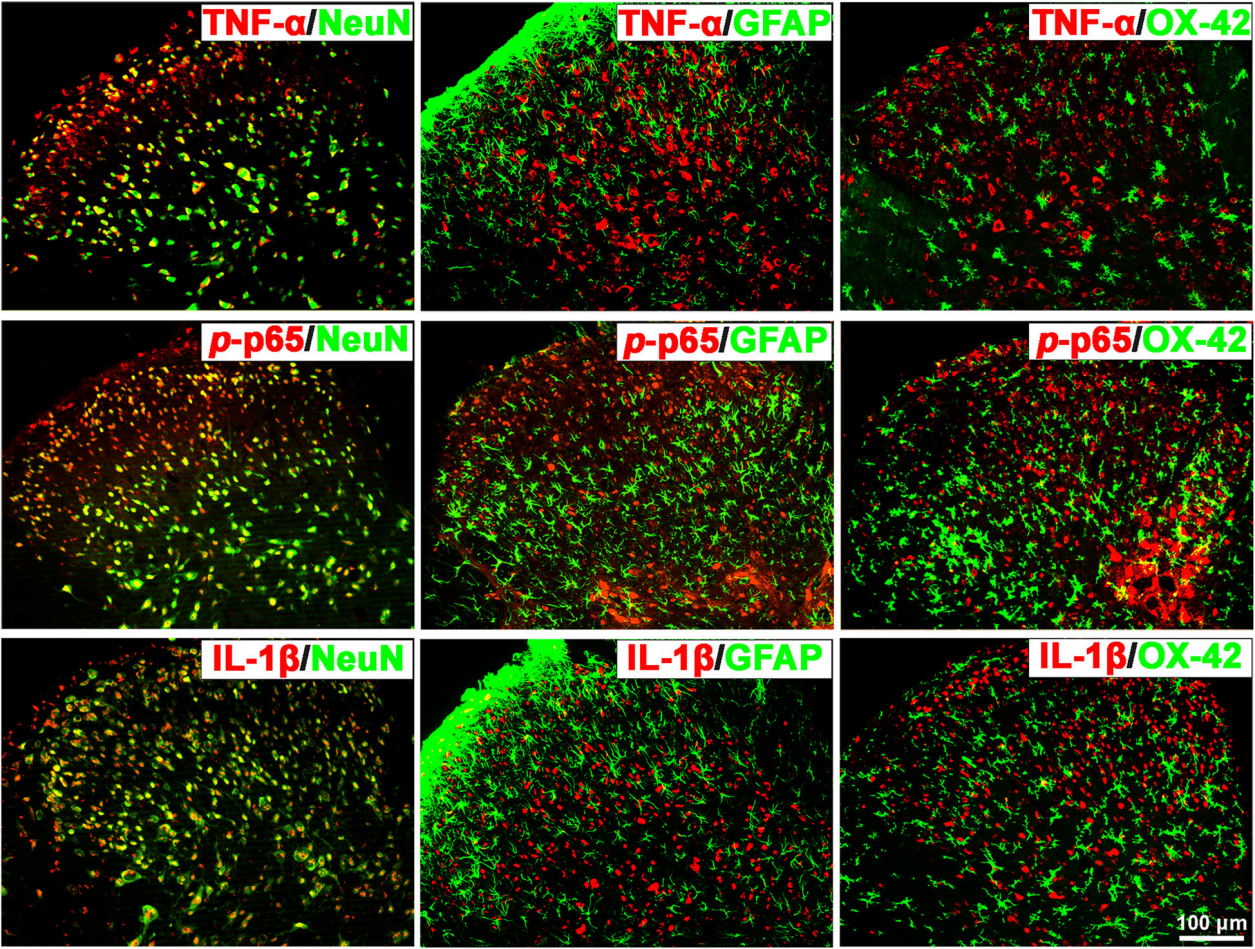
TNF-α, *p*-p65, and IL-1β all co-localize with NeuN in the SDH of CYP-induced cystitis rats. Photographs of the double immunofluorescence staining indicate that TNF-α, *p*-p65 (a principal transcriptional regulator of the activation of NF-kB pathway), and IL-1β were only co-localized with NeuN-labeled neurons but not with GFAP-labeled astrocytes or OX-42-labeled microglia in the SDH of cystitis animals

**Fig. 6:**
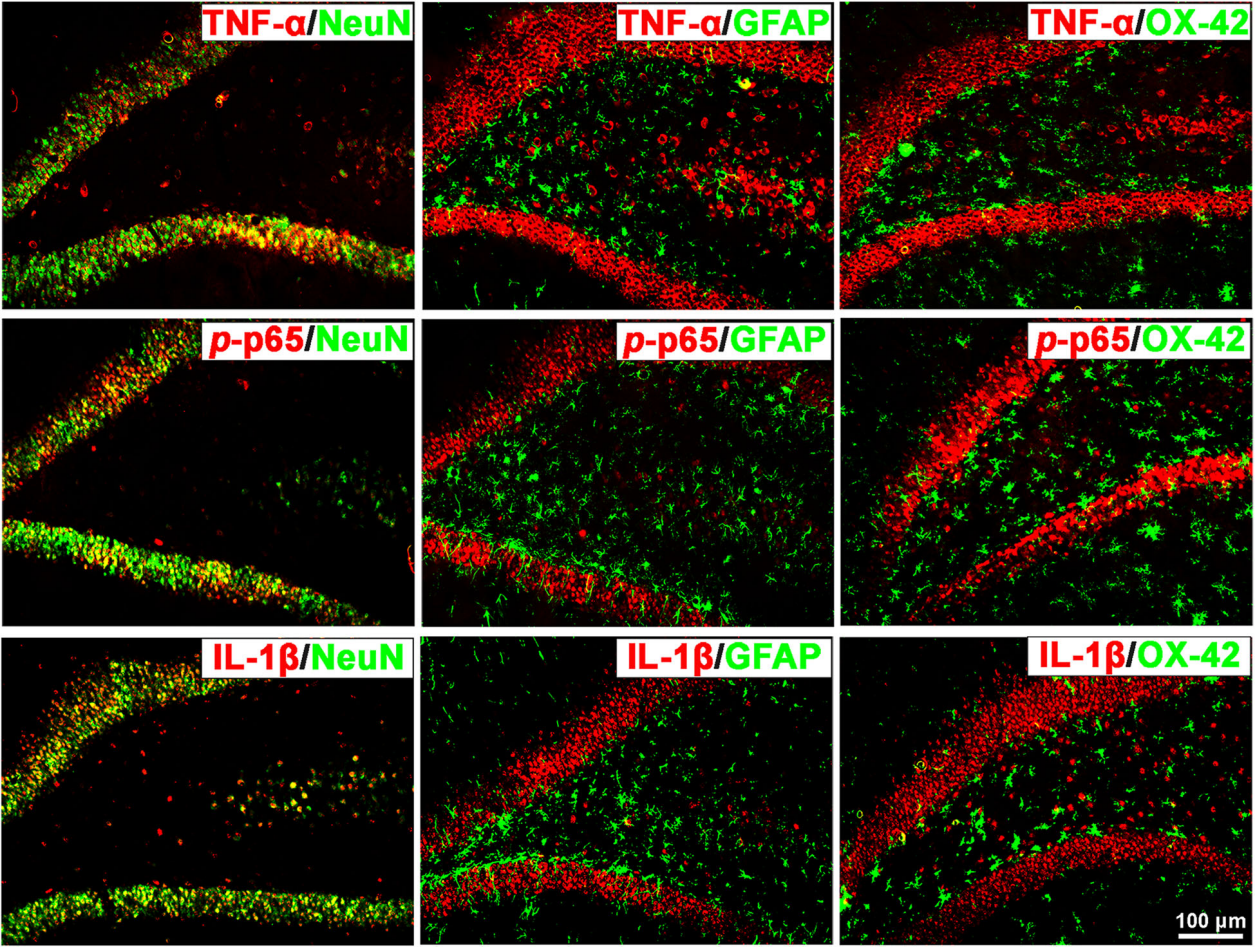
TNF-α, *p*-p65, and IL-1β co-localized with NeuN in the hippocampus of the CYP-induced cystitis model. Double immunofluorescence staining indicates that TNF-α, *p*-p65, and IL-1β were also co-localized with NeuN but not with GFAP or OX-42 in the hippocampus of cystitis animals

Furthermore, we explored the co-localization of TNF-α, *p*-p65 (a principal transcriptional regulator of the activation of the NF-kB pathway), and IL-1β in the SDH and hippocampus of cystitis model rats using double immunofluorescence staining. The results indicate that both in the SDH and hippocampus, all three proteins co-localized with NeuN-labeled neurons but not with GFAP-labeled astrocytes or OX-42-labeled microglia (Figs. 5 and 6).

**Fig. 7:**
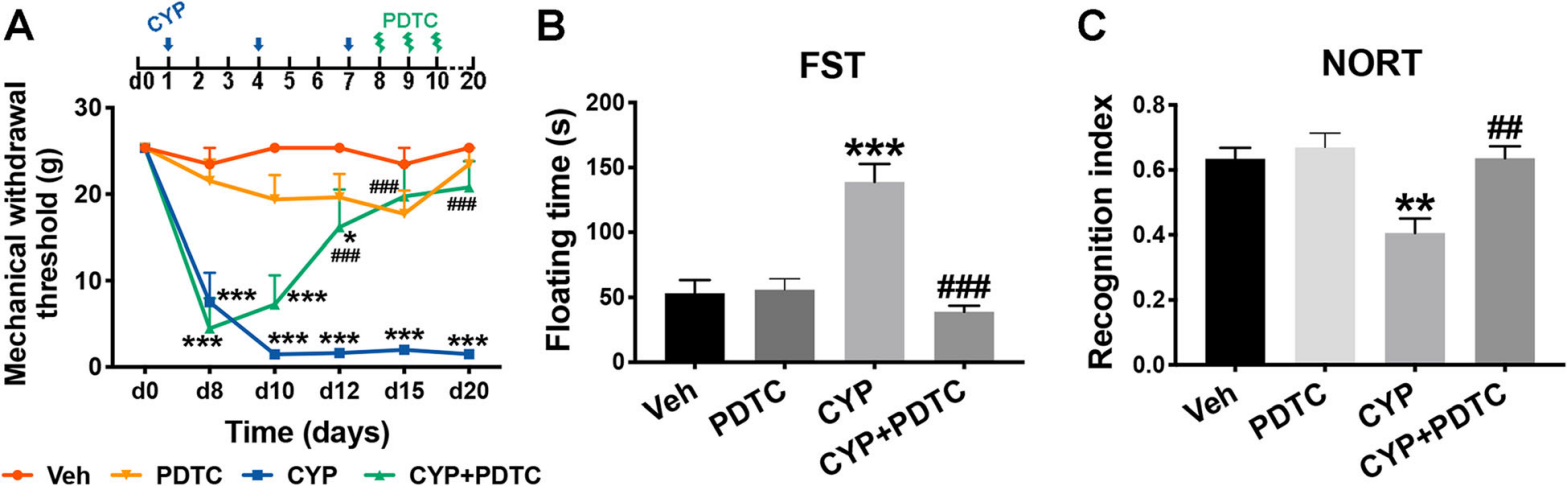
Inhibition of NF-κB signaling attenuated mechanical allodynia, depressive-like behavior, and STMD in CYP-induced cystitis model. **a** Intrathecal injection of pyrrolidinedithiocarbamate ammonium (PDTC), an inhibitor of NF-κB signaling, substantially attenuated mechanical allodynia from day 12 after the first CYP injection. After day 15, the mechanical withdrawal threshold of the CYP + PDTC group became non-significantly different from that of the vehicle group. The data were analyzed by two-way ANOVA followed by the Tukey post hoc test. **b** Treatment with PDTC significantly reversed the depressive-like behavior of cystitis rats, indicated by a longer floating time in the FST. **c** The recognition index in the NORT was reduced in cystitis rats, which was normalized by PDTC treatment. The data in **b**and **c** were analyzed by one-way ANOVA followed by the Tukey post hoc test. *n* = 7 per group in the von Frey test. *n* = 5 per group in the FST and NORT. **P < 0.01 and ***P < 0.001 vs. Veh group. ^##^*P* < 0.01 and ^###^*P* < 0.001 vs. CYP group

To further validate the role of pro-inflammatory NF-κB signaling in the mechanism of mechanical allodynia and comorbid depressive-like behaviors or STMD in the cystitis model, we used PDTC, an inhibitor of NF-κB signaling, to evaluate its influence on the behavioral test results. As Fig. 7 shows, intrathecal injection of PDTC (200 ng per day for three consecutive days) could reverse the mechanical allodynia induced by CYP-induced cystitis, as well as return pain-associated depressive-like behaviors and STMD to a basal level.

### Oral application of L-TAMS inhibits the upregulation of TNF-α/NF-κB signaling and IL-1β in the SDH and hippocampus of CYP-induced cystitis rats

A previous study indicated that L-TAMS attenuated vincristine-induced allodynia via inhibition of TNF-α/NF-κB signaling [38] in the SDH, and could restore memory deficits in spare nerve injury (SNI) rats through inhibition of TNF-α at a hippocampal level [14]. Combined with the upregulation of TNF-α/NF-κB signaling and IL-1β found in both the SDH and hippocampus of cystitis model rats, we hypothesized that oral application of L-TAMS could attenuate mechanical allodynia, memory deficits, and depressive-like behaviors through inhibition of TNF-α/NF-κB signaling and IL-1β in the SDH and hippocampus of cystitis rats.

**Fig. 8:**
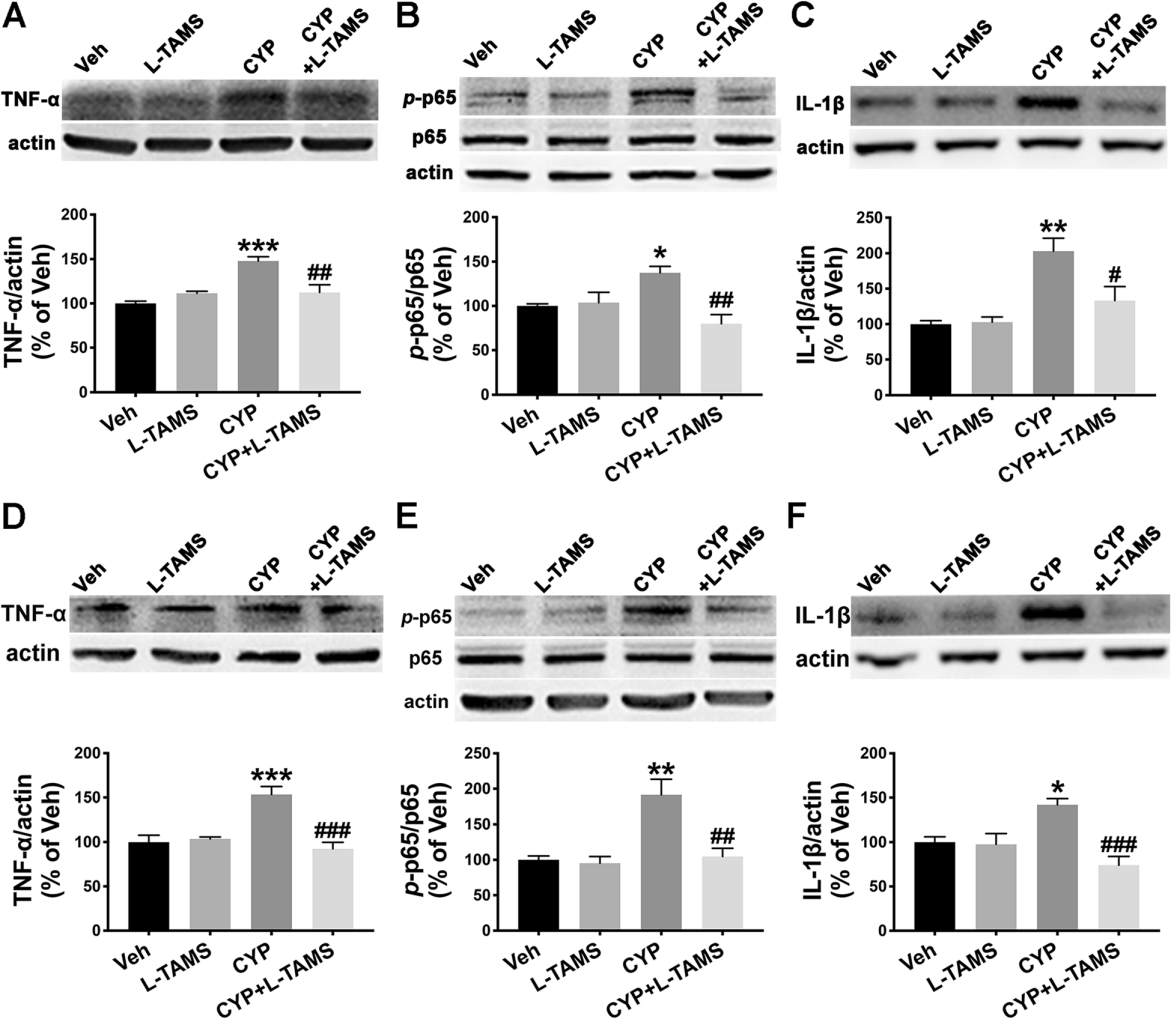
L-TAMS inhibited the TNF-α, *p*-p65, and IL-1β increase observed in the SDH and hippocampus. **a–c** L-TAMS inhibited the increased expression of TNF-α, *p*-p65, and IL-1β observed in the SDH of cystitis rats harvested on day 20 after the first CYP injection. **d**–**f** The increased expression of TNF-α, *p*-p65, and IL-1β in the hippocampus of cystitis model rats was also attenuated by L-TAMS treatment on day 20. *P < 0.05, **P < 0.01, and ***P < 0.001 vs. Veh group, ^#^*P* < 0.05, ^##^*P* < 0.01, and ^###^*P* < 0.001 vs. CYP group. All data were analyzed by one-way ANOVA followed by the Tukey post hoc test

**Fig. 9:**
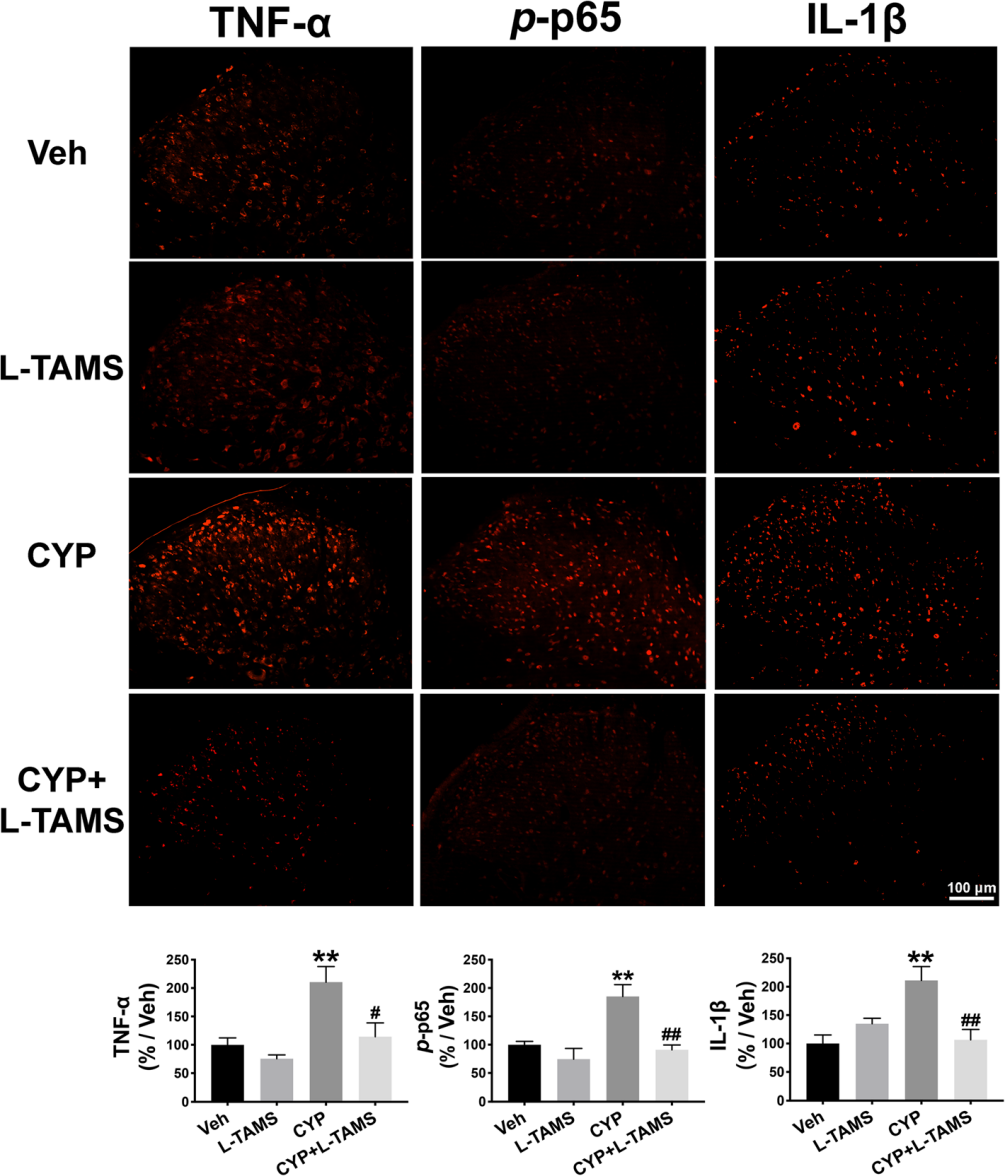
Increased expression of TNF-α, *p*-p65, and IL-1β in the SDH was reversed by L-TAMS. The immunofluorescence results show that TNF-α, *p*-p65, and IL-1β were all upregulated on day 20 after the first CYP injection, and this was significantly inhibited by L-TAMS treatment. No significant difference was observed between the CYP + L-TAMS group and Veh group. **P < 0.01 vs. Veh group, ^#^*P* < 0.05 and ^##^*P* < 0.01 vs. CYP group. Data were analyzed by one-way ANOVA followed by Tukey’s post hoc test

**Fig. 10:**
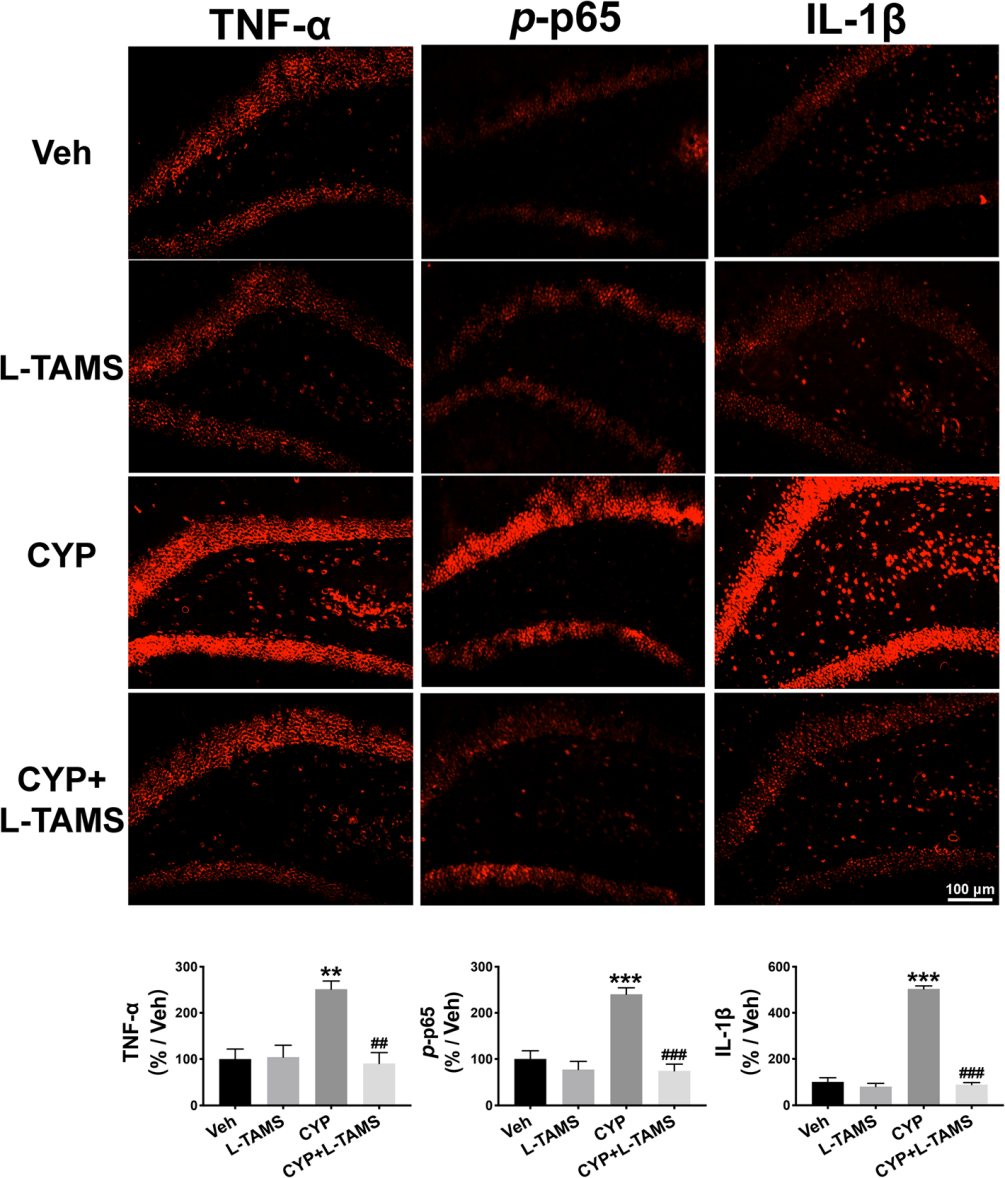
Upregulation of TNF-α, *p*-p65, and IL-1β in the hippocampus was reversed by L-TAMS. The expression of TNF-α, *p*-p65, and IL-1β were all found to be increased on day 20 after the first CYP injection. The expression of the three proteins in the cystitis group treated with L-TAMS was significantly lower than that in the untreated cystitis group. No significant difference was observed between the CYP + L-TAMS group and Veh group. **P < 0.01 and ***P < 0.001 vs. Veh group, ^##^*P* < 0.01 and ^###^*P* < 0.001 vs. CYP group. Data were analyzed by one-way ANOVA followed by Tukey’s post hoc test

Therefore, we performed a western blot analysis and immunofluorescence staining to determine TNF-α/NF-κB signaling as well as IL-1β expression in the SDH and hippocampus of CYP-induced cystitis rats and compared the expression level between groups with and without L-TAMS treatment. The results of the western blot analysis indicate that the upregulation of TNF-α, the *p*-p65/p65 ratio, and IL-1β in the SDH of the cystitis model following L-TAMS treatment was all significantly inhibited on day 20 when compared with the cystitis group without L-TAMS treatment (Fig. 8a–c). Moreover, as shown in Fig. 8d–f, overexpression of TNF-α, the *p*-p65/p65 ratio, and IL-1β in the hippocampus of cystitis animals was also prevented after L-TAMS treatment when compared to the group without treatment. The immunofluorescence results also show a similar change to that of the western blot analysis (Figs. 9 and 10). Furthermore, the western blot and immunofluorescence analyses both showed that the expression of TNF-α, IL-1β, and the *p*-p65/p65 ratio in the CYP + L-TAMS group became non-significantly different from that in the vehicle group. Additionally, the expression difference of TNF-α/NF-κB signaling and IL-1β between the vehicle and L-TAMS groups was non-significant.

### Up- and downregulation of NR2B in the SDH and hippocampus, respectively, were normalized by oral application of L-TAMS in the CYP-induced cystitis model

NMDA receptor (NMDAR), especially the NR2B subunit, plays a key role in synaptic plasticity. Activation of NR2B in the SDH is critically associated with neuropathic pain [39, 40]. However, the downregulation of NR2B in the hippocampus would impair synaptic plasticity contributing to memory deficits [41] and the downregulation has also been observed in the hippocampus of a rat model of depression [42, 43]. Besides, NR2B can be modulated by Mg^2+^ [44] as well as TNF-α [14]. Therefore, we speculated that expression of NR2B in the SDH and hippocampus of the CYP-induced cystitis model may have a similar opposite change at the two central nerve system levels, and magnesium supplementation with L-TAMS could normalize the change.

**Fig. 11:**
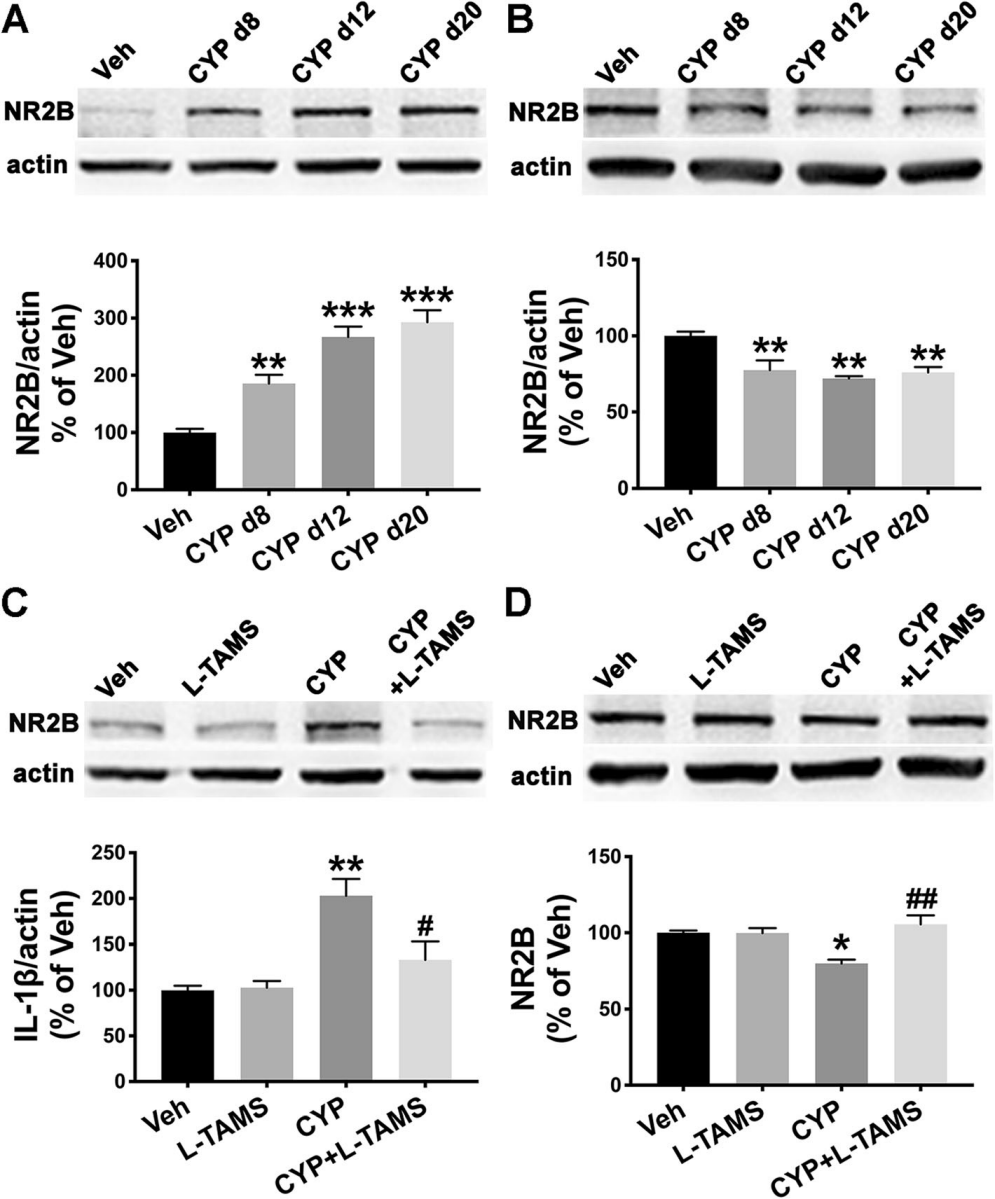
Up- and downregulation of NR2B in the SDH and hippocampus, respectively, were normalized by L-TAMS. Western blot analysis showed that NR2B was upregulated in the SDH (**a**) but downregulated in the hippocampus (**b**) of cystitis model rats at the three time points (days 8, 12, and 20 after the first CYP injection). The abnormal expression of NR2B in the SDH (**c**) or hippocampus (**d**) was neutralized by oral application of L-TAMS on day 20. *P < 0.05, **P < 0.01, and ***P < 0.001 vs. Veh group, ^#^*P* < 0.05 and ^##^*P* < 0.01 vs. CYP group. Data were analyzed by one-way ANOVA followed by Tukey’s post hoc test

We explored the expression of NR2B in the SDH and hippocampus of the cystitis model using western blot analysis. The result indicated that NR2B in the SDH was significantly upregulated at the three time points (Fig. 11a), whereas it was downregulated in the hippocampus at the three time points (Fig. 11b). Additionally, the overexpression of NR2B in the SDH was inhibited by L-TAMS application for 12 days, as assessed on day 20 following the first CYP injection (Fig. 11c), and the low-expression of NR2B in the hippocampus was also reversed on day 20 (Fig. 11d). We did not find any effect of L-TAMS on NR2B expression in normal rats (Fig. 11c, d).

## Discussion

In our present research, we revealed that there is a magnesium deficiency in both the serum and CSF of CYP-induced cystitis model rats. The magnesium deficiency was positively correlated with both mechanical allodynia and comorbid depressive-like behaviors and memory deficits. As a magnesium compound that can elevate brain magnesium by oral administration, L-TAMS was able to reverse the magnesium deficiency in the cystitis model and to attenuate the mechanical allodynia as well as the comorbid depressive-like behaviors and memory deficits. Neuroinflammation and synaptic plasticity play an important role in the mechanism of CYP-induced cystitis, and we found that TNF-α/NF-κB signaling and IL-1β were upregulated in both the SDH and hippocampus of the cystitis model. However, the NR2B subunit of NMDAR showed opposing expression at the two different central nerve system levels, showing greater expression in the SDH but less in the hippocampus. Surprisingly, oral application of L-TAMS could reverse and normalize the expression changes in TNF-α/NF-κB signaling, IL-1β, and NR2B in both the SDH and hippocampus of the CYP-induced cystitis model. Taken together, L-TAMS attenuated mechanical allodynia, depressive-like behavior, and memory deficits in CYP-induced cystitis model rats through prevention of magnesium deficiency and abnormal regulation of TNF-α/NF-κB signaling, IL-1β, and the NR2B subunit in the SDH and hippocampus.

### The role of magnesium deficiency in mechanical allodynia and the comorbid depressive-like behaviors and memory deficits in the cystitis model

As mentioned in the “Background” section, it is believed that patients with BPS/IC are at greater risk of having depression and memory problems. However, no research has revealed the mechanism underlying the comorbidity of bladder-related pain and depression or memory dysfunction in BPS/IC. Our present work did not focus on clarifying such a complex mechanism, but instead, we focused on establishing a common therapeutic target for both bladder-related pain and the accompanying depression and memory dysfunction. Such research could be of clinical value for the treatment of BPS/IC.

Magnesium deficiency could be an underlying cause accounting for both bladder-related pain and comorbid depression and memory dysfunction in BPS/IC. Previous studies showed that magnesium deficiency in rats, induced by a Mg-depleted diet, showed hyperalgesia, which could be attenuated by supplementation with MgSO_4_ [11, 12]. Moreover, magnesium deficiency is also associated with the neuropathic pain produced by diabetes [10, 45] and chemotherapy agents [24, 46]. Although visceral pain differs a lot from neuropathic pain, our present study showed magnesium deficiency in both the serum and CSF of cystitis rats. Furthermore, Mg^2+^ levels in the serum or CSF were positively correlated with the mechanical withdrawal threshold. Based on the above findings, supplementation of magnesium was taken into account to attenuate mechanical allodynia in the cystitis model. However, some basic and clinical research reported a negative effect of magnesium compounds on pain. One group demonstrated that oral administration of MgCI_2_ failed to reduce neuropathic pain in patients [47]. A significant antinociceptive effect of i.t. administration of MgSO_4_ was not observed in an acute pain model [48]. Additionally, the effect of the perioperative use of MgSO_4_ on postoperative pain is still controversial [49]. The unsatisfying elevation of magnesium levels in the CSF possibly accounts for the negative therapeutic outcome of MgCI_2_ and MgSO_4_. Research has indicated that the perioperative use of MgSO_4_ could not successfully elevate the magnesium concentration in the CSF and this was negatively correlated with cumulative postoperative analgesic consumption [50]. Likewise, MgCI_2_ injected i.p. was unable to change the brain magnesium level in rats [51]. Therefore, we intended to find a suitable magnesium compound that is more effective at elevating brain Mg^2+^. We therefore used L-TAMS, which has been validated to more efficiently transport Mg^2+^ into the central nervous system [22]. Our present work shows that oral application of L-TAMS was able to normalize the reduced Mg^2+^ level both in the serum and CSF of cystitis rats. More importantly, L-TAMS could attenuate the mechanical allodynia in cystitis rats, though pre-treatment could not prevent the onset of allodynia.

In addition, it has been well established that a magnesium-deficient diet leads to depressive behavior [52] and impairments in emotional memory [53] in normal mice. Moreover, magnesium treatment has been successfully used to treat major depression [54] and treatment-resistant depression [55]. Increasing brain magnesium using L-TAMS enhances short- and long-term memory in both young and aged rats [22]. As comorbidities of bladder-related pain, depression and memory deficits may share some common pathological mechanisms with the above conditions. Interestingly, we showed that magnesium deficiencies in both the serum and CSF were associated with depressive behavior or STMD in the cystitis model. The results share some points of similarity with a previous result showing that Mg^2+^ levels in serum were correlated with both pain scores and the depression index in patients with fibromyalgia [13]. Coincidently, the prevalence of fibromyalgia is much higher in patients with BPS/IC [56], indicating that the two syndromes may share common pathogenesis and pathophysiology. Our present work also showed that L-TAMS was able to normalize the depressive behavior and STMD in cystitis rats. The results further suggest that magnesium deficiency may contribute to the accompanying depression and memory deficits.

### The mechanisms by which normalization of magnesium deficiency attenuated mechanical allodynia and comorbidities

It is widely believed that NF-κB signaling is a critical proinflammatory pathway, and *p*-p65 is a principal transcriptional regulator of the activation of the NF-κB pathway [57]. TNF-α is a classical and canonical factor that stimulates the phosphorylation of p65 and helps it transfer to the nucleus where it activates numerous target genes, including TNF-α itself and IL-1β [58].

It has long been proposed that TNF-α/NF-κB signaling plays a crucial role in pathological pain [59]. Additionally, TNF-α/NF-κB signaling has also been shown to be involved in the development of depression [60] and the regulation of memory [61]. Therefore, the question is, can TNF-α/NF-κB signaling act as a promotor in the mechanism of comorbidities of pain such as depression or memory deficits? Research has indicated that during the development of chronic neuropathic pain, persistent noxious stimuli could result in an increase in TNF-α in the hippocampus, which then disrupts the depression-related brain circuit of hypothalamic-pituitary-adrenal axis dysfunction [36]. Overexpression of TNF-α in the hippocampus was also revealed to lead to neuropathic pain and memory deficits in the SNI model [62]. Additionally, TNF-α can potentiate positive feedback to produce more TNF-α through the NF-κB pathway, worsening the seriousness of pain and the depressive-like symptoms and memory deficits. Based on the above evidence, we hypothesize that TNF-α/NF-κB signaling is an underlying mechanism of bladder-related pain and comorbid depressive-like behaviors and memory impairment in the cystitis model. The results in our present study showed that TNF-α/NF-κB signaling was activated and more IL-1β was produced at both the spinal cord and hippocampal level. Furthermore, inhibition of the NF-κB pathway showed a therapeutic effect on mechanical allodynia and comorbid depressive-like behaviors or STMD, validating the role of NF-κB signaling in the mechanism common to the comorbidities of pain and depressive-like behaviors or memory dysfunction in the cystitis model.

Next, we explored whether TNF-α/NF-κB signaling is associated with magnesium deficiency in vivo. A previous study provides evidence that magnesium-deficient animals exhibit a generalized inflammatory tone induced by elevated circulating levels of the proinflammatory cytokines TNF-α and IL-1β [20]. Aged animals also present with similar increases in proinflammatory cytokines [20]. In our present work, reversal of magnesium deficiency with L-TAMS normalized the upregulation of TNF-α/NF-κB signaling and the associated increase in IL-1β. The result is somewhat consistent with that in the vincristine-induced pain [24], SNI [14], and AD [63] models. Taken together, upregulation of TNF-α/NF-κB signaling and the associated increase in IL-1β are underlying downstream mechanisms of magnesium deficiency in the CYP-induced cystitis model.

Synaptic plasticity is an important mechanism involved in the regulation of pain, depression, and memory function. TNF-α is not only a promotor of neuroinflammation, but also a regulator of synaptic plasticity. A previous study showed that overproduction of TNF-α can induce long-term potentiation (LTP) in the SDH [64] but inhibit LTP in the hippocampus [37] of a nerve injury model, which ultimately leads to pain and memory deficits, respectively. NMDARs, especially NR2B subunit-containing NMDARs, are believed to be activators of LTP. In our present work, we did not directly examine LTP in our cystitis model, but instead focused on the expression of the NR2B subtype. Interestingly, up- and downregulation of NR2B were observed in the SDH and hippocampus, respectively, of the cystitis model rats. The region-dependent differences in NR2B expression is in accordance with reports in many chronic pain conditions [65]. Upregulation of TNF-α in both the SDH and hippocampus could account for such differences, leading to spinal LTP and hippocampal LTD, respectively. Furthermore, it has been proposed that magnesium is an essential modulator of NMDAR. Therefore, Mg^2+^ could represent a target to modulate NR2B expression in the cystitis model. Surprisingly, our work revealed that L-TAMS could normalize rather than increase or decrease NR2B expression in the SDH and hippocampus. This result is consistent with that in the vincristine-induced allodynia model. We consider the opposing change in NR2B expression as an underlying mechanism of mechanical allodynia and memory deficits in cystitis rats, accounting for the therapeutic effect of L-TAMS on both mechanical allodynia and memory deficits of cystitis rats through normalization of NR2B expression. However, this hypothesis should be validated with more research.

Last but not least, TNF-α/NF-κB signaling and IL-1β participate in many biological activities, including immunity, cell proliferation, differentiation, and apoptosis [66, 67], and as mentioned above, the NR2B subunit of NMDAR is an important modulator of synaptic plasticity. Accordingly, it is quite important to maintain physiological levels to prevent any side effects. In our present work, L-TAMS did not affect the expression of the aforementioned proteins in normal animals. Interestingly, after treatment with L-TAMS, the abnormal expression of proteins in cystitis rats were all reversed to the level seen in vehicle-treated animals. Thus, L-TAMS may represent a suitable means to treat BPS/IC.

The main limitation of this study is that we only focused on two central nervous system regions, the SDH and hippocampus, to carry out the research on whether and how normalization of magnesium deficiency by L-TAMS plays a role in the cystitis model. This limits our ability to clarify an integrated mechanism of comorbidities in the central nervous system. A further limitation is that we only investigated mechanisms in the hippocampus in this study, though many brain regions including the hippocampus, prefrontal cortex, and amygdala are involved in the initiation and development of nociceptive sensitization, depression, and memory deficits, which are complex processes. More research on the change in circuits among different brain regions in the cystitis model is needed to clarify such processes. Moreover, we need to further investigate the downstream mechanism of magnesium deficiency evoking bladder-related pain, depression, and memory deficits. Lastly, our results might have been affected by the estrus cycle phase of the examined female animals.

## Conclusions

Magnesium deficiency in the serum and CSF is associated with mechanical allodynia, depressive-like behaviors, and memory deficits in the CYP-induced cystitis model. Moreover, TNF-α/NF-κB signaling and pro-inflammatory IL-1β were both upregulated in the SDH and hippocampus of the cystitis rats. However, expression of the NR2B subunit showed changes in opposing directions in the SDH and hippocampus. Oral application of L-TAMS substantially reversed the magnesium deficiency and upregulation of TNF-α/NF-κB signaling and IL-1β and normalized the expression of NR2B. In turn, this led to the attenuation of mechanical allodynia, depressive-like symptoms, and memory deficits in the CYP-induced cystitis model. Our study revealed that magnesium supplementation with L-TAMS could be of great clinical value for the treatment of BPS/IC.

## Supplementary information

**Additional file 1: Table S1.** Number of animals used in each behavioral test and molecular experiment (per group). **Table S2.** Antibody information. **Figure S1.** Identification of specificity of the antibody for *p*-p65 used in our study. The specificity of the antibody for *p*-p65 was identified by pre-absorption with *p*-p65 (S311) blocking peptide provided by the manufacturer.

## Data Availability

The authors should be contacted if any data or material is required to be provided.

## Abbreviations

BPS: Bladder pain syndrome
CSF: Cerebrospinal fluid
CYP: Cyclophosphamide
FST: Forced swim test
GFAP: Glial fibrillary acidic protein
IC: Interstitial cystitis
IL-1β: Interleukin-1 beta
L-TAMS: Magnesium-L-Threonate
NeuN: Neuronal nuclei
NF-κB: Nuclear factor-κB
NORT: Novel object recognition test
NR2B: *N*-Methyl-d-aspartate receptor type 2B subunit
PDTC: Pyrrolidinedithiocarbamate ammonium
SDH: Spinal dorsal horn
SPT: Sucrose preference test
STMD: Short-term memory deficits
TNF-α: Tumor necrosis factor-α
