# Correction to: Normalization of magnesium deficiency attenuated mechanical allodynia, depressive-like behaviors, and memory deficits associated with cyclophosphamide-induced cystitis by inhibiting TNF-α/NF-κB signaling in female rats

**DOI:** 10.1186/s12974-021-02258-0

**Published:** 2021-11-14

**Authors:** Jia-Liang Chen, Xin Zhou, Bo-Long Liu, Xu-Hong Wei, Hong-Lu Ding, Zhi-Jun Lin, Hai-Lun Zhan, Fei Yang, Wen-Biao Li, Jun-Cong Xie, Min-Zhi Su, Xian-Guo Liu, Xiang-Fu Zhou

**Affiliations:** 1 Department of Urology, The Third Affiliated Hospital of Sun Yat-sen University, 600 W Tianhe Rd, Guangzhou, 510630 China; 2 Pain Research Center and Department of Physiology, Zhongshan School of Medicine, Sun Yat-sen University, 74 Zhongshan Rd. 2, Guangzhou, 510080 China; 3 Guangdong Provincial Key Laboratory of Brain Function and Disease, 74 Zhongshan Rd. 2, Guangzhou, 510080 China; 4 Department of Rehabilitation, The Third Affiliated Hospital and Lingnan Hospital of the Sun Yat-sen University, 2693 Kaichuang Rd., Guangzhou, 510700 China

**Correction to: J Neuroinflammation 17, 99 (2020)**

**https://doi.org/10.1186/s12974-020-01786-5**

Following publication of the original article [1], the authors noticed some mistakes in the published article as follows:

1. Methods, Drug administration:
  “Briefly, CYP (25 mg/kg; Sigma, St Louis, MO) was intraperitoneally...” should be corrected as following “Briefly, CYP (50 mg/kg; Sigma, St Louis, MO) was intraperitoneally...”. The dose of CYP we used in this research should be 50 mg/kg (same as described in Abstract) but not 25 mg/kg.

2. The histogram in Fig. 11c is mistakenly used. It is the same as the histogram in Fig. 8c. The Y axis title of Fig. 11d should be corrected to “NR2B/actin (% of Veh)”. Presented here is the corrected Fig. 11.

**Fig. 11:**
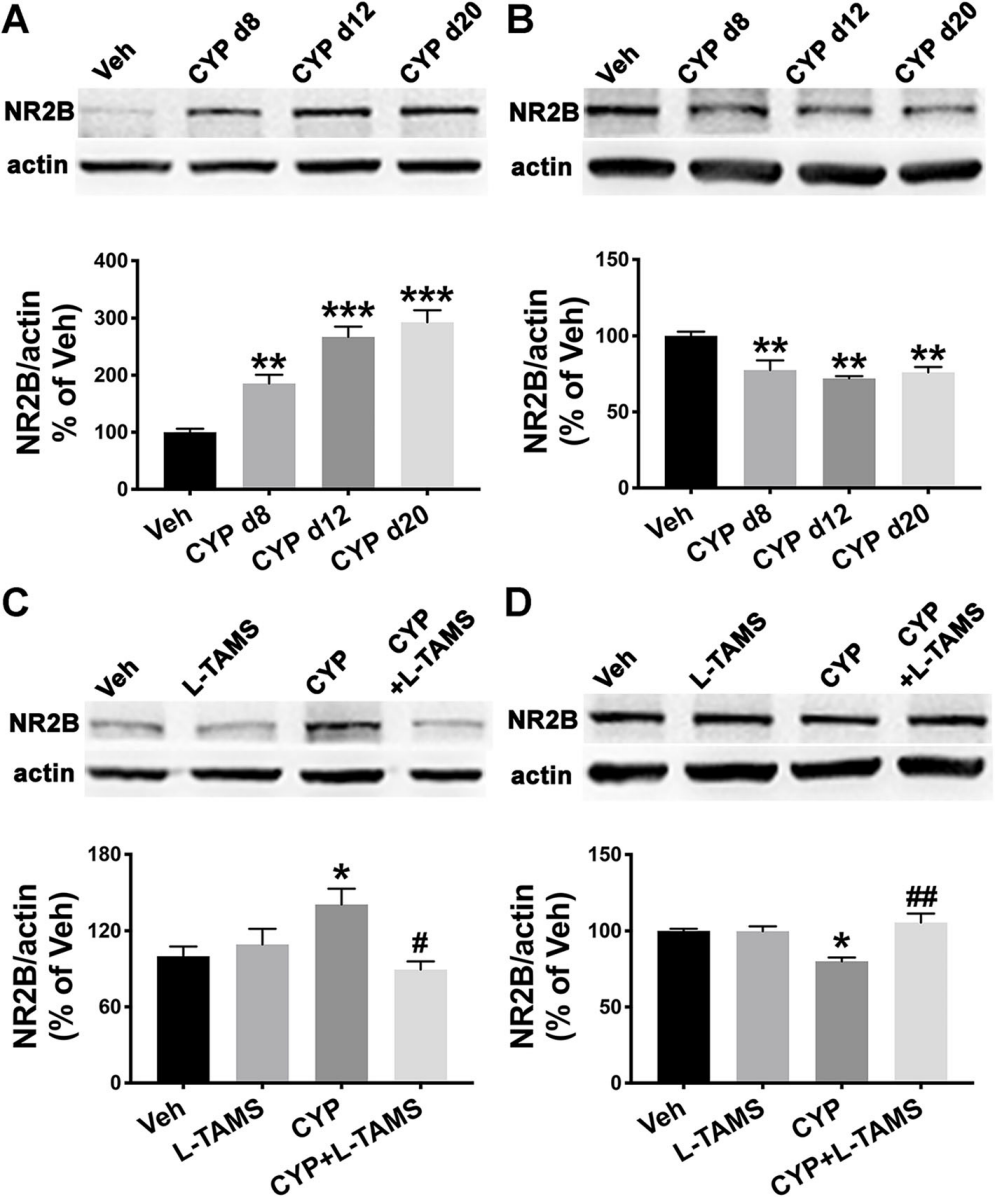
Up- and downregulation of NR2B in the SDH and hippocampus, respectively, were normalized by L-TAMS. Western blot analysis showed that NR2B was upregulated in the SDH (**a**) but downregulated in the hippocampus (**b**) of cystitis model rats at the three time points (days 8, 12, and 20 after the first CYP injection). The abnormal expression of NR2B in the SDH (**c**) or hippocampus (**d**) was neutralized by oral application of L-TAMS on day 20. *P < 0.05, **P < 0.01, and ***P < 0.001 vs. Veh group, ^#^*P* < 0.05 and ^##^*P* < 0.01 vs. CYP group. Data were analyzed by one-way ANOVA followed by Tukey’s post hoc test

The original article has been updated.
