# Supplementary material for: Normalization of magnesium deficiency attenuated mechanical allodynia, depressive-like behaviors, and memory deficits associated with cyclophosphamide-induced cystitis by inhibiting TNF-α/NF-κB signaling in female rats
Source: J Neuroinflammation. 2020 Apr 2;17:99. doi: 10.1186/s12974-020-01786-5 (PMC7118907; doi:10.1186/s12974-020-01786-5)
Supplement: Supplementary file 1 — Additional file 1: Table S1. Number of animals used in each behavioral test and molecular experiment (per group). Table S2. Antibody information. Figure S1. Identification of specificity of the antibody for p-p65 used in our study. The specificity of the antibody for p-p65 was identified by pre-absorption with p-p65 (S311) blocking peptide provided by the manufacturer. [file 12974_2020_1786_MOESM1_ESM.docx]

## Normalization of magnesium deficiency attenuated mechanical allodynia, depressive-like behaviors, and memory deficits associated with cyclophosphamide-induced cystitis by inhibiting TNF-α/NF-κB signaling in female rats

**Supplemental Table 1. Number of animals used in each behavioral test and molecular experiment (per group)**

|  | Pain measurement | NORT | FST | SPT | WB | IF | Mg^2+^ measurement | Total |
| --- | --- | --- | --- | --- | --- | --- | --- | --- |
| Part I | - | - | - | - | 5 | - | 5 | 10 |
| Part II | 10 | 10 | 10 | 5 | 5 ^1^ | 4 ^1^ | 9-10 ^2^ | 35 |
| Part III | 7 | 5 | 5 | 5 | - | - | - | 22 |

Annotation: NORT, novel object recognition test; FST, forced swim test; SPT, sucrose preference test; WB, western blot; IF, immunofluorescence

^1^ After pain measurement

^2^ After each behavioral test, 10 for serum and 9 for CSF

**Supplemental Table 2. Antibody information**

| Antibody | Supplier | IDENTIFIER/REFERENCE |
| --- | --- | --- |
| Rabbit anti-TNF-α | Bioworld Technology | Cat# BS1857, RRID: AB_1662107 |
| Rabbit anti-*p*-p65 | Affinity Biosciences | Cat# AF3389 |
| Rabbit anti-p65  Rabbit anti-IL-1β  Rabbit anti-NR2B  Rabbit anti-β-actin  Mouse anti-NeuN  Mouse-anti-GFAP  Mouse-anti-OX-42 | Abcam  Abcam  Abcam  Cell Signaling Technology  Millipore  Cell Signaling Technology  Abcam | Cat# ab16502, RRID:AB_443394  Cat# ab9722, RRID:AB_308765  Cat# ab65783, RRID:AB_1658870  Cat# 4967, RRID:AB_330288  Cat# MAB377, RRID:AB_2298772  Cat# 3670, RRID:AB_561049  Cat# ab1211, RRID:AB_442947 |


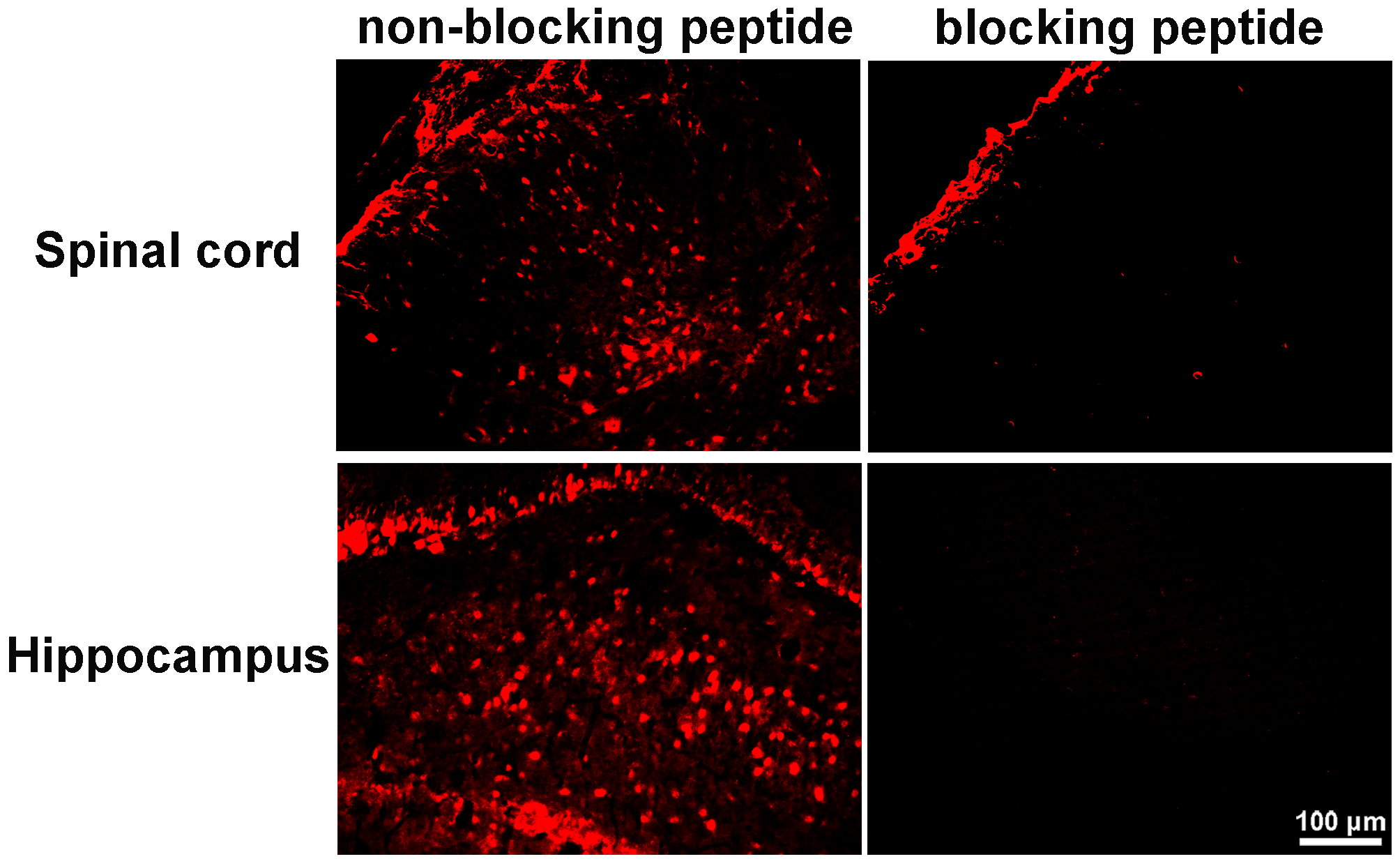


**Supplemental Figure 1. Identification of specificity of the antibody for *p*-p65 used in our study.** The specificity of the antibody for *p*-p65 was identified by pre-absorption with *p*-p65 (S311) blocking peptide provided by the manufacturer.
